# Assessment of balance and risk for falls in a sample of community-dwelling adults aged 65 and older

**DOI:** 10.1186/1746-1340-14-3

**Published:** 2006-01-27

**Authors:** Cheryl Hawk, John K Hyland, Ronald Rupert, Makasha Colonvega, Stephanie Hall

**Affiliations:** 1 Vice President of Research and Scholarship, Cleveland Chiropractic College, Kansas City, MO, and Los Angeles, CA; 2 Research Associate, Research Institute, Parker College of Chiropractic, Dallas, TX; 3 Director of Research, Research Institute, Parker College of Chiropractic, Dallas, TX; 4 Assistant Professor, Research Institute, Parker College of Chiropractic, Dallas, TX; 5 Research Assistant, Research Institute, Parker College of Chiropractic, Dallas, TX

## Abstract

**Background:**

Falls are a major health concern for older adults and their impact is a significant public health problem. The chief modifiable risk factors for falls in community-dwellers are psychotropic drugs, polypharmacy, environmental hazards, poor vision, lower extremity impairments, and balance impairments. This study focused on balance impairments. Its purpose was to assess the feasibility of recruiting older adults with possible balance problems for research conducted at a chiropractic research center, and to explore the utility of several widely used balance instruments for future studies of the effect of chiropractic care on balance in older adults.

**Methods:**

This descriptive study was conducted from September through December 2004. Participants were recruited through a variety of outreach methods, and all were provided with an educational intervention. Data were collected at each of two visits through questionnaires, interviews, and physical examinations. Balance was assessed on both visits using the Activities-specific Balance Confidence Scale (ABCS), the Berg Balance Scale (BBS), and the One Leg Standing Test (OLST).

**Results:**

A total of 101 participants enrolled in the study. Advertising in the local senior newspaper was the most effective method of recruitment (46%). The majority of our participants were white (86%) females (67%). About one third (32%) of participants had a baseline BBS score below 46, the cut-off point for predicting risk of falling. A mean improvement in BBS scores of 1.7 points was observed on the second visit. For the subgroup with baseline scores below 46, the mean change was 4.5 points, but the group mean remained below 46 (42.5).

**Conclusion:**

Recruitment of community-dwelling seniors for fall-related research conducted at a chiropractic research center appears feasible, and the most successful recruitment strategies for this center appeared to be a combination of targeted newspaper ads and personal contact through senior centers. The BBS and OLST appear to be promising screening and assessment instruments, which might have utility in future investigations of the possible effects of chiropractic care on balance.

## Background

Falls are one of the major health care concerns for older adults and their impact is a significant public health problem. Annually, about one-third of community-dwellers over age 65 fall, and half of those will have a repeat fall[1-3]. Falls are responsible for two-thirds of all unintentional injury deaths in older adults[4,5]. Fear of falling affects confidence in performing daily activities, causing self-limitation and a less active lifestyle[6]. This results in muscle atrophy and loss of strength, especially in the lower extremities, which exacerbates the risk for falls[7]. Direct and indirect costs associated with falls total $75–100 billion in the U.S. annually[5,8].

The most important modifiable risk factors for falls in community-dwelling older adults are use of psychotropic drugs, polypharmacy, environmental hazards, poor vision, lower extremity impairments, and impairments in balance, gait and activities of daily living[5]. This array of contributing causes makes the prevention of falls complex, requiring a multidisciplinary approach[9]. Because of their clinical focus on the neuromusculoskeletal system, chiropractors' scope of practice is congruent with the services of geriatric health care teams. At this time, however, there is very little evidence that chiropractic care, specifically spinal manipulation, has any influence on balance, one of the important modifiable risk factors for falls[10-12]. The purpose of this preliminary study was to assess the feasibility of recruiting older adults with possible balance problems for research conducted at a chiropractic research center, and to explore the utility of several widely used balance instruments for future studies of the effect of chiropractic care on balance in older adults.

## Methods

### Overview and specific aims

This was a descriptive study conducted in a chiropractic research center located in a large metropolitan area from September through December 2004, with a convenience sample of approximately 100 volunteers aged 65 and older.

The study's specific aims were to:

1) assess the feasibility of recruiting patients to our research center for a study of the effect of chiropractic care on balance problems in people aged 65 and older;

2) describe our sample of community-dwelling adults aged 65 and older, in terms of demographics, health history, medication use, and health habits;

3) conduct an intervention consisting of providing participants with a booklet of instructions on balance exercises and a home hazard checklist; and

4) compare participants 4 weeks after baseline to assess changes in balance scores as measured by the Berg Balance Scale, One Leg Standing Test and the Activities-specific Balance Confidence scale.

### Study population and eligibility criteria

#### Inclusion criteria

Community-dwelling ambulatory volunteers aged 65 and older who agreed to participate and signed the informed consent were eligible. Volunteers who required an assistive device (cane or walker) to walk were eligible.

#### Exclusion criteria

Potential participants were excluded if they were:

1) wheelchair-bound; this precluded required balance testing

2) unable to stand unassisted for a minimum of 1 minute; this precluded required balance testing

3) non-English-speaking, this precluded understanding verbal instructions since we had no translators available.

### Human subjects issues and informed consent

The study was approved by the college's Institutional Review Board prior to recruitment. Informed consent was obtained verbally and in writing from all participants. Eligible volunteers who completed both visits were compensated $50 for their time and travel.

### Recruitment

Participants were recruited through: 1) posters, 2) word-of-mouth, 3) newspaper advertisements, 4) presentations at senior centers and events, 5) radio advertisements, 6) college clinic/intern referrals, and 7) website advertisements. Modifications in the recruitment process were consistently made to reflect the success or failure of each recruitment strategy. Problems in recruitment methods were discussed at weekly team meetings and potential solutions were implemented.

*Posters *were placed in college's outpatient clinics, library, highly visible campus locations, and local Dallas senior centers. The posters were printed in a variety of vibrant colors designed to attract the viewer, using the header "Are you well-balanced?"

*Word-of mouth *recruitment through friends and family members was considered to be a potentially useful method, since many of our participants appeared to lead active and sociable lifestyles. At the end of the initial clinic visit, participants were asked to voluntarily distribute information and colorful printed hand-outs to eligible friends and family, and members of church and activity groups.

*Ads in local senior newspapers *also used the header "Are you well-balanced?" These were placed in a popular local senior publication as a means of reaching a greater population base of seniors. This publication was circulated to all senior centers and senior health organizations in the Dallas metropolitan area.

*Presentations at senior centers and events *were frequently utilized as a method of making personal contact with potential participants. An internet search of senior activity centers located in the entire metropolitan area was conducted using the following keywords: "senior centers," senior retirement centers," "senior recreation center," and "senior social center." Anticipating potential problems with senior transportation, a list was generated of contacts in locations that did not exceed a 15–20 mile radius. The research coordinator contacted the recreation/activity director of each facility and gave information about the study with a request to present the information to the residents. Announcements for senior events in a local senior publication were examined and event coordinators were contacted for a potential booth to display our information.

*Radio advertisement*s were used minimally. One radio station whose listener base consists mainly of older people was used for approximately 2–3 weeks of periodic advertisements. No cost was incurred since a former patient provided the service for free as a public service announcement.

*Clinic/Intern referrals *were facilitated by the location of the Research Institute adjacent to the clinic, enhanced by an informational session provided by study personnel to the clinic personnel and interns. Since participants would not be receiving chiropractic care in this study, there was no potential competition for patients with the clinic.

*Website Announcements *on the college's main website were provided at no cost and provided accessibility to a large population, including college employees, students, and the general public.

### Study period

The study included two visits – baseline and four weeks after the baseline visit.

### Data collection and assessment

Data were collected at each of the two visits, through self-report questionnaires, Research Assistant (RA) interviews and physical assessments and examinations performed by RAs. Compliance with exercise recommendations and home hazard checklists was assessed by self report; the RA questioned the patient during the interview and recorded his or her response. With respect to the exercise recommendations, the RA asked each patient: "Did you do any of the balance exercises we gave you at the first visit?" and gave the patient the options of "not at all," "occasionally," or "regularly," with definitions of these terms left up to the participant.

*Demographics, health history, and history of falls*. Questionnaires, both self-report and interviews were designed based on forms used in previous studies and included demographics, health history (including medication use), health habits and history of falls. For the history of falls, the RA defined "fall" to the patient as "accidentally ending up on the floor or ground."

Physical exam measures included height, weight, and blood pressure. Patients were asked to bring all their current medications with them at their first visit and the RA recorded them. We also included two questions from the Behavioral Risk Factors Surveillance Survey (BRFSS) concerning "healthy days"[13].

#### Balance assessments

1) *The Activities-specific Balance Confidence Scale (ABC scale) *has been shown to be predictive of falls in the elderly[14]. It is a 16-item questionnaire completed by the patient that inquires about their self-confidence in performing various activities of daily living that require balance. Scores range from 0–100.

2) *The Berg Balance Scale (BBS) *is a 14-item functional test involving common actions (e.g. sit to stand, picking up an object, standing on one leg) necessary for performing activities of daily living. Participants were scored on a 5-point (0–4) ordinal scale depending on their ability to complete the requested action[15]. A score of 0 was assigned when the task could not be completed, and a score of 4 indicated independence. The reliability and validity of the BBS in assessing balance have been documented, both in nursing home and community-dwelling older adults[16], and it is an effective predictor of falls within community-dwelling adults[17]. A score of 45 or less is used by most investigators to indicate a greater risk for falls[18-20]. Research assistants were trained to perform the BBS according to standard protocols as described in the literature and with advice from a physical therapist familiar with the BBS.

3) *The One Leg Standing Test (OLST) *is a commonly used balance assessment of postural stability among physical therapists and occupational therapists. Patients are given specific instructions to stand on one leg for as long as possible in one of two conditions, with the eyes open or eyes closed. Times are then recorded for the duration that the position was held. The OLST demonstrates moderate to high interrater and test-retest reliability based on time when used with adults (but not in children under the age of 9)[21,22]. The OLST is considered to be potentially useful in predicting functional decline, and has been shown to be sensitive to clinical interventions[22,23].

### Educational intervention materials

All participants received a package of printed materials, including: a *home hazard booklet *based on information from the National Center for Injury Prevention and Control of the Centers for Disease Control and Prevention[24], a *leaflet on general dietary recommendations *such as increasing fruits, vegetables, fiber and fluids, and a *home exercise routine *focusing on balance [25]. The exercises were based on recommendations from the National Institute on Aging regarding exercises for older adults to improve balance[25]. The exercises were detailed in an attractive illustrated pamphlet, using large font and including a self-test for one leg standing. In addition, any participants who were tobacco users were provided with informational materials about cessation[26]. (Although tobacco use is not directly related to the purpose of this study, inclusion of these materials was our center's standard practice, which is consistent with national recommendations that all health care providers should provide counseling to tobacco users.)

### Data management and analysis

Data were entered into an SPSS (Version 12.0 for Windows) database. Quality control was performed by the principal investigator by reviewing hard-copy forms for completeness, running validation checks and verifying a minimum of 10% of electronic entries.

Descriptive statistics were computed to assess the specific aims. To assess possible changes in participants' balance, BBS, OLST, and ABC scores at baseline and follow-up were compared using a paired sample t-test.

## Results

### Recruitment, enrollment and attrition

As shown in Table I, advertising in the local senior newspaper supplied almost half (46%) of the participants. The smallest proportion of participants was recruited through radio advertisements (2%). Word-of-mouth and clinic/intern referrals contributed 16% and 11% of participants, respectively. Participants recruited from the word-of-mouth method frequently reported the major influence for their participation in this study to be the positive comments of satisfaction with the research staff expressed by their referring friends or family members. Several walk-in participants (10%) enrolled as a result of referrals from other participants; that is, they visited the research facility with friends or family members who were already enrolled.

**Table 1:** Recruitment resources

**Resource**	**% (n = 101)**
Ad in local senior newspaper	46
Word-of-mouth	16
College clinic/intern referral	11
Referral from study participants	10
Senior center presentations	7
College employee or employee relative	6
Unknown	3
Radio ads	2

Although recruitment from presentations at senior centers yielded approximately 7% of the participants, it proved to be an invaluable networking resource. Activity directors often requested that information about our study be faxed to them to display in their centers and refer participants. Transport was arranged for some participants by one senior center, and on those occasions the research center provided lunch.

Recruitment costs were as follows: 1) materials for posters and flyers, approximately $10; 2) travel and time for research staff preparing materials, making presentations at senior centers and talking with directors on the phone (estimated by an examination of schedules and calendars), approximately 25 person-hours, which is equivalent to approximately $500; 3) the chief cost was the $50 compensation provided to all participants who completed the study for time and travel; this totaled $4700.

A total of 101 participants were enrolled in the study; 94 completed both visits (93%). Explanations for the 7 participants who did not return involved the following: separation from husband combined with a loss of interest in the study (1), conflict with work schedule (2), scheduling problems with a social group leader in an ethnic community who wanted to bring in a group of non-English speaking people (1), lack of transportation (1), debilitating illness (1), and unknown reason/no response (1).

Of the 94 participants who completed both visits, 79% (74) said they were interested in participating in a future study involving chiropractic care for balance problems. Considering only the 26 participants with a baseline BBS score <45, 23 expressed an interest in participating in the future study.

### Sample characteristics

The majority of participants were female (67%), white (86%) and the average age was just over 73 years (Table 2). Participants were well-educated, with 94% having at least a high school diploma and 70% having at least some college education. Only 25% were retired, with 13% still employed full time.

**Table 2:** Participant demographics

**Characteristic**	**% (n = 101)**
*Gender*	
Female	67
Male	33
Mean age in years (range)	73.3 (65–91) SD = 6.5
*Marital status*	
Married or living with partner	45
Widow/widower living alone	25
Single/divorced living alone	30
*Race/ethnicity*	
White	86
Black/African American	5
Asian/Pacific Islander	5
Hispanic	4
American Indian	0
Mixed race	0
*Educational level*	
Did not complete high school	6
High school diploma	24
Some college	29
College degree	21
Post-graduate degree	14
Professional school	7
*Employment*	
Employed full-time	13
Employed part-time	62
Retired	25

All numbers are expressed as percents, unless otherwise specified

Our participants reported very healthy lifestyles; only 2% reported current tobacco use and 3% daily alcohol use (Table 3). Most reported engaging in some form of regular exercise, with over half reporting exercising 3 or more times each week.

**Table 3:** Participant health habits

**Health habit**	**% (n = 101)**
Mean cups of water consumed daily (range)	6.8 (0–20)
*Tobacco use*	
Currently use	2
Formerly used	46
Never used	53
*Alcohol use*	
Use daily	3
Use occasionally	38
Formerly used, not now	21
Never used	39
Mean cups of caffeine consumed daily (range)	2.1 (0–10)
*Aerobic exercise*	
Never	22
1–2 times/week	30
3+ times/week	48
*Exercise other than aerobic*	
Never	18
1–2 times/week	44
3+ times/week	38
*Functional assistance*	
Glasses/contacts most of time	75
Hearing aid	9
Need assistance to walk (cane, walker, support from guardrail or companion)	
Sometimes	11
Most of the time	9

All numbers are expressed as percents, unless otherwise specified

As shown in Table 4, we found a potential for depression, but very little disability in this group of community-dwelling seniors. The median number of days participants reported having restricted activity due to poor mental or physical health was 0; 71% reported 0 days. Many of the participants experienced musculoskeletal symptoms, with 53% reporting arthritis and 43% reporting low back pain. Those who reported having low back pain had significantly (p = .003) fewer days when they felt healthy and full of energy (entire question is shown in Table 4), although there was no difference in their days of restricted activity, compared to those without low back pain. The same observation held true for those reporting arthritis. Many reported other health conditions commonly associated with aging, including hypertension (35%), osteoporosis in the women (34%), prostate problems in the men (27%) and diabetes in both sexes (15%).

**Table 4:** Participant health status

	**% (n = 101)**
*Depression screeners (in last 2 weeks):*	
Felt down, depressed or hopeless	19
Felt little interest or pleasure in doing things	20
Have trouble sleeping	48
*BRFSS questions:*	
"During the past 30 days, for about how many days did poor physical or mental health keep you from doing your usual activities, such as self-care, work, or recreation?"	mean – 2.6 days median – 0 days range – 0–30 days
"During the past 30 days, for about how many days have you felt very healthy and full of energy?"	mean – 19.7 days median – 25 days range – 0–30 days
*Musculoskeletal conditions (in last month):*	
Arthritis	53
Low back pain	43
General joint pain or stiffness	43
Hip, leg and/or knee pain	43
Neck pain and/or stiffness	34
Muscle aches	27
Foot and/or ankle pain	21
Headache	17
Tingling or numbness of leg or foot	17
Upper back pain	14
Sciatica	5
*Other health conditions(in last month):*	
Hypertension	35
Osteoporosis	
Women (n = 68)	34
Men (n = 33)	3
Prostate problems (men only, n = 33)	27
Fatigue	24
Dizziness	20
Hearing impairment	18
Poor and/or blurred vision	17
Diabetes	15
*Medication use:*	
Prescription drugs	mean – 3.6 median – 3.0 range – 0–12
Nonprescription drugs	mean – 2.7 median – 2.0 range – 0–12

All numbers are expressed as proportions, unless otherwise specified

### Medication use

About one-third of patients (32%) forgot to bring their medications with them, so their medication use was self-reported rather than recorded directly by the RA. Fourteen percent of participants reported taking no prescription medications and 12% reported taking no nonprescription medications or vitamins and other supplements. The mean number of prescription medications was 3.6 (median 3.0) and nonprescription medications was 2.7 (median 2.0). Participants who used fewer than 4 medications per day had a baseline BBS score of 48.8, while those who used 4 or more medications per day had a baseline BBS score of 45.2. For those reporting concurrent use of more than 4 medications, the most commonly reported medications were allergy relief, cholesterol-lowering and anti-hypertension drugs.

### Falls and balance

Of the 101 participants, 13% reported having had a fall within the past month, and 44% within the last year. The average number of reported falls for the last year was 0.8; the median number was 0.

For participants' baseline BBS, 32% had a score less than 46 (the cut-off point for predicting risk of falling). Four weeks after the baseline visit, changes in balance test scores for all participants were statistically significantly improved for the BBS (mean change 1.7 points), OLST on the right and the ABC scale questionnaire (Table 5). As shown in Table 6, for those participants with a baseline BBS score less than 46, although the mean BBS score change was 4.5 (Table 6), the group mean remained below 46 at the follow-up visit.

**Table 5:** Comparison of mean scores on balance tests between baseline and 4-week follow-up visit for all participants (n = 94).

**Measure**	**Pre-test**	**Post-test**	**Mean difference**	**Significance**
Berg Balance Scale^1^	47.1 (17–56)	48.8 (13–56)	1.7	.001
Single leg standing (R)	13.1 (0–109)	18.6 (0–149)	5.5	.009
Single leg standing (L)	9.9 (0–138)	12.6 (0–120)	2.8	.147
ABC Scale	80.5 (23–100)	82.3 (25–100)	2.1	.034

Means were compared using a paired samples t-test. For all tests, a higher score indicates better function.

^1 ^For the Berg Balance Scale, n = 93; one patient did not perform this test at the follow-up due to an acute episode of dizziness.

**Table 6:** Comparison of mean scores on balance tests between baseline and 4-week follow-up visit for participants with baseline BBS scores < 46 (n = 32).

**Measure**	**Pre-test**	**Post-test**	**Mean difference**	**Significance**
Berg Balance Scale^1^	38.0 (17–45)	42.5 (13–56)	4.5	.001
Single leg standing (R)	2.4 (0–14)	6.6 (0–48)	4.3	.015
Single leg standing (L)	1.7 (0–5)	3.9 (0–30)	2.2	.035
ABC Scale	67.2 (23–99)	72.8 (25–100)	5.6	.016

Means were compared using a paired samples t-test. For all tests, a higher score indicates better function.

^1 ^For the Berg Balance Scale, n = 31; one patient did not perform this test at the follow-up due to an acute episode of dizziness.

### Use of balance exercises and home hazard checklist

The majority (72%) of the 94 participants who completed the follow-up visit reported that they had done the balance exercises regularly; 26% occasionally; and 2% not at all. Over half (60%) said they had gone over the home hazard checklist at home, and of those 56 people, 18 said they had been able to fix any of the fall hazards they identified. Viewing the BBS and OLST change scores by grouping participants by those who regularly did the balance exercises vs. those who occasionally/never did them, there was 0.3 point difference between the groups' BBS change scores (p = .799), 5 seconds difference between change scores for left leg standing (p = .223) and 9 seconds for right leg standing (p = .067).

## Discussion

The pragmatic aim of this study was to assess the feasibility of recruiting older adults into studies at our research center. Our results indicate that this population is willing to participate in research conducted at a chiropractic research center, and that the best way to publicize studies is through targeted ads combined with personal contact. Furthermore, attrition for this two-visit study was low (7%); participants were enthusiastic and amenable to the educational intervention.

While the study was quite successful in terms of recruiting participants, our results should not be generalized to other populations or geographical locations. There are several methodological limitations that affect our ability to draw conclusions from the data collected. First, much of the information collected was self-reported and based on recall, so descriptions of participants' activities, health habits and health events (such as falls), as well as medication use, are susceptible to these biases. Second, the absence of a comparison group necessitates caution in interpreting the observed improvements in the balance assessments. Third, the observed improvements may be statistically, but not clinically, significant. It was beyond the scope of this study to investigate the issue of clinical significance, particularly in terms of the effect of these observed improvements on risk for falls.

Although this sample of men and women aged 65 and older reported very healthy lifestyles, with little tobacco use, and inclusion of regular exercise, the proportion reporting no limitations on their daily activities was somewhat lower than the national average for people aged 65 and older (71% vs. 83%, respectively, using the most recent BRFSS data, which was from 2001[27]). It is interesting to note that low back pain and arthritis – conditions for which many patients seek chiropractic care – were associated with decreased days of feeling healthy and full of vitality but not with increased days of limitation of daily activities. Several patients indicated that they did not let pain limit them from doing things they needed or wanted to do.

Our participants' medication use may be a risk factor for balance impairment. One study found an increased risk for balance impairment for people who used 3–4 medications per day (OR 1.72)[28]. Providing some support for this, we found a somewhat lower baseline BBS for those who used 4 or more medications per day compared to those who used fewer than 4 medications per day (45.2 vs. 48.8, respectively).

The recommended balance exercises were well-accepted by participants, with 72% saying they had done the exercises regularly. Although we did not formally investigate the impact the exercises might have on balance, the BBS scores did not improve dramatically, although there was a slight suggestion that the OLST might show some response. The hazard checklist did not appear to be particularly effective in helping patients modify home hazards.

Although statistically significant improvements were seen in the mean scores for all measures of balance, with larger improvements among the subsample of patients with baseline scores < 46 (cut-off for fall risk), these improvements may not be clinically significant. Since the mean baseline BBS score for this sample was higher than 46, indicating fairly high function in terms of balance, it is likely that the mean improvement of less than 2 points was not clinically significant. Even in the subsample with a mean baseline BBS score lower than 46, the mean improvement of 4.5 points was not sufficient to raise the follow-up mean score above the cut-off point of 46.

It should also be noted that we cannot determine which, if any, study-related activities might have influenced the improvement in the balance test scores. Regular performance of the exercises did not, in our informal analysis of it, seem to have a strong relationship to the balance scores. It is possible that there was a simple learning effect operating in repeating the balance tests on the second visit. It is also possible that regression to the mean was present, particularly since the subsample with much lower baseline scores showed a greater improvement.

Future studies should further examine the role of the OLST as a test for balance and risk for falls, since it is a much simpler and faster method than the BBS. We are currently investigating the effect of chiropractic manipulation/adjustments on BBS scores among samples of older adults with self-reported balance problems.

## Competing interests

All authors declare that they have no financial or non-financial competing interests. No external funds or grants were used for this study.

## Authors' contributions

CH designed the study, wrote the proposal, analyzed the results and contributed to writing the paper. JKH, RR, MC, and SH contributed to the study design, interpretation of the results and writing of the paper. MC recruited participants and coordinated the project. SH did the data management. CH and JKH led the writing of the paper, and all authors read and approved the final manuscript.
